# Sub-Lineage Specific Phenolic Glycolipid Patterns in the *Mycobacterium tuberculosis* Complex Lineage 1

**DOI:** 10.3389/fmicb.2022.832054

**Published:** 2022-03-08

**Authors:** Nicolas Gisch, Christian Utpatel, Lisa M. Gronbach, Thomas A. Kohl, Ursula Schombel, Sven Malm, Karen M. Dobos, Danny C. Hesser, Roland Diel, Udo Götsch, Silke Gerdes, Yassir A. Shuaib, Nyanda E. Ntinginya, Celso Khosa, Sofia Viegas, Glennah Kerubo, Solomon Ali, Sahal A. Al-Hajoj, Perpetual W. Ndung’u, Andrea Rachow, Michael Hoelscher, Florian P. Maurer, Dominik Schwudke, Stefan Niemann, Norbert Reiling, Susanne Homolka

**Affiliations:** ^1^Bioanalytical Chemistry, Priority Area Infections, Research Center Borstel, Leibniz Lung Center, Borstel, Germany; ^2^Molecular and Experimental Mycobacteriology, Priority Area Infections, Research Center Borstel, Leibniz Lung Center, Borstel, Germany; ^3^Department of Microbiology, Immunology, and Pathology, Colorado State University, Fort Collins, CO, United States; ^4^Lung Clinic Grosshansdorf, Airway Disease Center North (ARCN), German Center for Lung Research (DZL), Großhansdorf, Germany; ^5^Municipal Health Authority Frankfurt am Main, Frankfurt am Main, Germany; ^6^Municipal Health Authority Hannover, Hanover, Germany; ^7^College of Veterinary Medicine, Sudan University of Science and Technology, Khartoum, Sudan; ^8^WHO-Supranational Reference Laboratory of Tuberculosis, Institute of Microbiology and Laboratory Medicine (IML Red), Gauting, Germany; ^9^National Institute for Medical Research Tanzania – Mbeya Medical Research Center, Mbeya, Tanzania; ^10^Instituto Nacional de Saúde (INS), Marracuene, Mozambique; ^11^Department of Medical Microbiology and Parasitology, School of Medicine, Kenyatta University, Nairobi, Kenya; ^12^Department of Microbiology, Immunology, and Parasitology, St. Paul’s Hospital Millennium Medical College, Addis Ababa, Ethiopia; ^13^Mycobacteriology Research Section, Department of Infection and Immunity, King Faisal Specialist Hospital and Research Centre, Riyadh, Saudi Arabia; ^14^Institute of Tropical Medicine and Infectious Diseases (ITROMID), Jomo Kenyatta University of Agriculture and Technology (JKUAT), Nairobi, Kenya; ^15^Division of Infectious Diseases and Tropical Medicine, University Hospital, LMU Munich, Munich, Germany; ^16^German Centre for Infection Research (DZIF), Partner Site Munich, Munich, Germany; ^17^National and WHO Supranational Reference Centre for Mycobacteria, Research Center Borstel, Leibniz Lung Center, Borstel, Germany; ^18^Institute of Medical Microbiology, Virology, and Hygiene, University Medical Center Hamburg-Eppendorf, Hamburg, Germany; ^19^German Center for Infection Research (DZIF), Partner Site Hamburg-Lübeck-Borstel-Riems, Borstel, Germany; ^20^Airway Research Center North, Member of the German Center for Lung Research (DZL), Borstel, Germany; ^21^Microbial Interface Biology, Priority Area Infections, Research Center Borstel, Leibniz Lung Center, Borstel, Germany

**Keywords:** *Mycobacterium tuberculosis* complex, single nucleotide polymorphism (SNPs), NMR, phenolic glycolipids, genetic diversity, phylogeny, whole genome sequencing (WGS), structural chemistry

## Abstract

“Ancestral” *Mycobacterium tuberculosis* complex (MTBC) strains of Lineage 1 (L1, East African Indian) are a prominent tuberculosis (TB) cause in countries around the Indian Ocean. However, the pathobiology of L1 strains is insufficiently characterized. Here, we used whole genome sequencing (WGS) of 312 L1 strains from 43 countries to perform a characterization of the global L1 population structure and correlate this to the analysis of the synthesis of phenolic glycolipids (PGL) – known MTBC polyketide-derived virulence factors. Our results reveal the presence of eight major L1 sub-lineages, whose members have specific mutation signatures in PGL biosynthesis genes, e.g., *pks15/1* or glycosyltransferases Rv2962c and/or Rv2958c. Sub-lineage specific PGL production was studied by NMR-based lipid profiling and strains with a completely abolished phenolphthiocerol dimycoserosate biosynthesis showed in average a more prominent growth in human macrophages. In conclusion, our results show a diverse population structure of L1 strains that is associated with the presence of specific PGL types. This includes the occurrence of mycoside B in one sub-lineage, representing the first description of a PGL in an *M. tuberculosis* lineage other than L2. Such differences may be important for the evolution of L1 strains, e.g., allowing adaption to different human populations.

## Introduction

The genetic diversity of *Mycobacterium tuberculosis* complex (MTBC) strains has been shown to influence transmission and virulence of clinical MTBC isolates as well as the immune response and clinical outcome (reviewed in Coscolla and Gagneux, 2014; Tientcheu et al., 2017). MTBC strains of so called “modern” phylogenetic lineages (clade 1) (Wirth et al., 2008; Barbier and Wirth, 2016) are defined by a genomic deletion known as TbD1 (Brosch et al., 2002). Clade 1 strains that comprises Lineage 2 (L2, Beijing genotype), Lineage 3 [L3, Delhi/Central-Asian (Delhi/CAS)], and Lineage 4 (L4, Euro-American) are responsible for the majority of tuberculosis (TB) cases worldwide (Brites and Gagneux, 2017). In contrast, the distribution of patients infected with clade 2 strains (“ancestral” strains defined by an intact TbD1 region) including Lineage 1 [L1, East African Indian genotype (EAI)], Lineage 5 [L5, *M. africanum* West African 1 genotype (WA1)], and Lineage 6 [L6, *M. africanum* West African 2 genotype (WA2)] (Wirth et al., 2008) are geographically more restricted and show a globally lower prevalence in comparison to “modern” strains (Brites and Gagneux, 2017). Especially, L1 strains are a prominent cause of TB on the Indian subcontinent (Gutierrez et al., 2006; Thomas et al., 2011) and in Malaysia (Ismail et al., 2014). It has been reported that L1 strains display a reduced transmission rate in comparison to “modern” strains of L2 or L4 (Albanna et al., 2011; Nebenzahl-Guimaraes et al., 2015). One of the molecular reasons for that is linked to the presence of the TbD1 region in L1 strains. Loss of TbD1 in “modern” MTBC strains has been associated with a better circumvention of and persistence against oxidative stress and hypoxic conditions within the host cell micro-environments. This may generate an important advantage for those MTBC pathogens especially during prolonged stages of infection (Bottai et al., 2020). In addition, these observations are in line with data from own experiments using *in vitro* and *in vivo* model systems, since we previously showed that “modern” MTBC strains from L2 or L4 exhibit significantly enhanced growth rates in human monocyte-derived and alveolar macrophages as well as in aerogenically infected mice, when compared to L1 strains (Reiling et al., 2013).

Cell wall components such as lipids and lipoglycans including trehalose dimycolates (TDM), sulfolipids (SL), phthiocerol dimycocerosates (PDIM) and phenolic glycolipids (PGL; also termed glycosyl phenolphthiocerol dimycoserosates) have been discussed as virulence factors of MTBC strains (Forrellad et al., 2013; Madacki et al., 2019).

Trehalose dimycolates, also known as the mycobacterial cord factor, plays a major role in modulating the acute response of the host cell upon entry of the MTBC pathogen. It has been shown that TDM modulates the early M1-like macrophage response as seen during initiation of the granulomas of primary pathology (Nguyen et al., 2020). Furthermore, TDM delays phagosomal maturation through MINCLE signaling (Axelrod et al., 2008; Patin et al., 2017) and antagonizes IFNγ-induced expression of MHC-II as well as antimicrobial effector genes such as GBP1 (Huber et al., 2020).

Independent studies have shown that PDIM and also structurally related PGL are lipid virulence factors that are associated with mycobacterial host evasion and modulate the host immune response in many ways (Quigley et al., 2017; Mishra et al., 2019; Bah et al., 2020). *Mycobacterium tuberculosis* mutants showing PDIM transport deficiency induced in THP-1 macrophages lower levels of microtubule-associated protein light chain 3 (LC3), often used as autophagy marker associated with the macrophage membranes, when compared to the respective wild-type strain (CDC1551) (Quigley et al., 2017). Mishra et al. (2019) proposed a multifaceted role of SL, since SL-1 modulates key biophysical properties of the host cell membrane such as fluidity, hydration and lipid domain re-organization. Bah et al. (2020) showed that SL limits TLR/MyD88-dependent and phagosomal damage-independent autophagy by acting as a Toll-Like Receptor 2 (TLR2) antagonist, whereas PDIM prevents this process to a certain extent by favoring Esx-1-dependent phagosomal damage. Furthermore, it was shown that PDIM, but not SLs, limit the acidification of LC3-positive compartments containing *M. tuberculosis*. Finally, PDIM also mask other pathogen associated molecular pattern on the cell wall, thereby dampening immune responses mediated by pattern recognition receptors (Arbues et al., 2014; Cambier et al., 2014).

**FIGURE 1:**
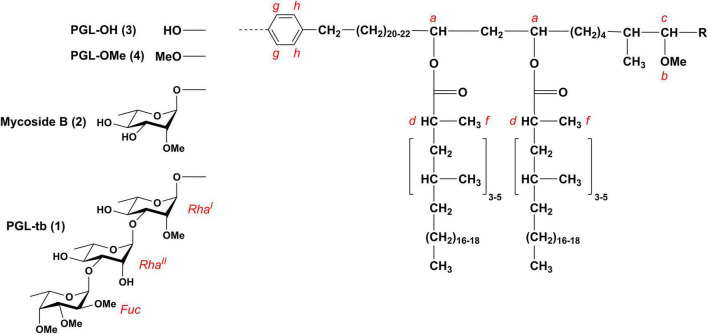
Chemical structures of PGL-tb (**1**), mycoside B (**2**), and phenolphthiocerol dimycoserosates [PGL-OH (**3**)/PGL-OMe (**4**)]. Composition of the lipid core and the letter code are adapted from Pérez et al. (2004); R = CH_2_–CH_3_ or CH_3_.

Phenolic glycolipids are capable of recruiting permissive macrophages via chemokine receptor 2 enabling the pathogen to reach the lower respiratory tract (Cambier et al., 2014, 2017). PGL have further been shown to inhibit the TLR2 response, leading to the reduction of pro-inflammatory cytokine production (Arbués et al., 2016; Blanc et al., 2017). A subset of L2 clinical isolates displayed a hypervirulent phenotype in mice, which was correlated with the presence of PGL-tb [2,3,4-tri-*O*-methyl-L-fucopyranosyl-α-(1→3)-L-rhamnopyranosyl-α-(1→3)-2-*O*-methyl-L-α-(1→)-rhamnopyranosyl phenolphthiocerol dimycoserosate (**1**); Figure 1; Reed et al., 2004]. Although PGL-tb (**1**) is capable of modulating the early host cytokine response (Stadthagen et al., 2006) and contributes to the manipulation of macrophage recruitment by the pathogen as described above, (Cambier et al., 2014) it does not confer hypervirulence itself. A biochemically engineered PGL-tb (**1**)-producing H37Rv strain, a strain normally devoid of PGL-tb (**1**) synthesis, suppressed the induction of several pro- and anti-inflammatory cytokines *in vitro* in human monocytes, similar to PGL-tb (**1**)-producing clinical *M. tuberculosis* isolates. However, this did not lead to increased virulence in infected mice and rabbits (Sinsimer et al., 2008). In contrast to L2 strains, all strains of the Euro-American lineage (L4) lack PGLs and phenolphthiocerol dimycoserosates (phenolic PDIM) due to a frameshift in the *pks15/1* gene (Constant et al., 2002). Notably, this frameshift does not affect the presence of PDIM (Constant et al., 2002; Siméone et al., 2010). Moreover, *M. tuberculosis* strains that are unable to produce PGL-tb (**1**) due to this frameshift can still produce glycosylated *para*-hydroxybenzoic acid methyl esters (pHBAD), which are essential building blocks for PGL biosynthesis (Pérez et al., 2004). Such molecules have been shown to dampen the immune response as well (Stadthagen et al., 2006; Bourke et al., 2014). Interestingly, a mutation in Rv2962c (Q294Stop), which deletion leads to an abolishment of PGL biosynthesis at the stage of phenolic PDIM (Pérez et al., 2004), was described in strains of the L1 lineage (Indo-Oceanic lineage) (Krishnan et al., 2011). However, the impact on pathogenicity mediated by the molecular type of PGL (Figure 1) synthesized in the MTBC strains, either as PGL-tb (**1**), mycoside B (**2**), phenolphthiocerol dimycoserosates PGL-OH (**3**) and PGL-OMe (**4**), is not yet fully elucidated (Arbues et al., 2014; Barnes et al., 2017).

This study aimed to link the population structure of L1 strains to the presence of distinct PGL types and to address their potential correlation with differences in virulence. First, we analyzed the global population structure of L1 strains by Whole Genome Sequencing (WGS) analysis of 312 strains from 43 countries. Based on this L1 population structure, we determined major sub-lineages, out of which representative clinical isolates were selected for further analysis of their lipid composition using NMR-based approaches and performed growth analyses in human macrophages, the natural host cell of the TB pathogen.

## Materials and Methods

### Bacterial Strains and Growth Conditions

Bacterial strains were initially cultured from clinical samples on Löwenstein/Jensen (L/J) media at the National Reference Center for Mycobacteria in Borstel, Germany. The MTBC clinical isolates used in this study were handled to minimize *in vitro* passaging. All cultures used in this study were derived from frozen stocks prepared after a single *in vitro* passage of original archived samples. The virulent lab strain H37Rv ATCC 27294, however, has undergone countless rounds of *in vitro* passaging. All strains were further characterized by WGS as previously described and phenotypic susceptibility testing according to WHO standards (Meehan et al., 2019).

Homogenous bacterial suspensions were prepared from L/J cultures in 10 ml 7H9 media (Difco Becton Dickinson, Sparks, MD, United States) supplemented with 10% OADC (Becton Dickinson GmbH, Heidelberg, Germany), 0.05% Tween 80 (Merck KGaA, Darmstadt, Germany) as well as 0.2% glycerol (Merck KGaA, Darmstadt, Germany) and incubated in 30 ml square media bottles (Nalgene^®^, Thermo Fisher Scientific, Darmstadt, Germany) at 37°C without shaking (pre-culture). Growth to mid-log phase was monitored by measuring OD_600_ every second day (BioTek Synergy2, Agilent, Santa Clara, CA, United States). Pre-cultures were transferred into a roller bottle system (Corning^®^, Merck KGaA, Darmstadt, Deutschland) and incubated at 37°C and 5 rpm. 20 ml of fresh media were gradually added to a final volume of 100 ml (main-culture). Mid-log phase bacteria suspension (OD_600_ 0.2–0.4) were stored in 1 ml aliquots at –80°C for further investigations. Colony forming units were determined for each strain investigated. Furthermore, sterility controls (Ziehl-Neelsen staining, blood agar, LB media, BHI bouillion) were performed for all pre- and main-cultures.

For ^1^H, ^13^C-HSQC NMR lipid profiling an aliquot of a given MTBC strains was thawn, centrifuged at 4,500 × *g* and the supernatant was discarded. The bacteria were resuspended in 1 ml PBS using a G 26 × 1/2′′/ø 0.45 mm × 12 mm Sterican^®^ canula (B. Braun, Melsungen, Germany) and inoculated in 25–40 ml 7H9 media with the addition of 10% self-prepared OADC containing ^13^C glucose (D-Glucose U-^13^C_6_, Euriso-Top, Gif-sur-Yvette, France), 0.2% ^13^C_3_ glycerol (Cambridge Isotope Laboratories, Andover, MA, United States) and 0.05% Tyloxapol (Sigma-Aldrich Chemie GmbH, Steinheim, Germany).

Bacteria were grown in roller bottles at 37°C until an OD_600_ of 0.6–0.8. Subsequently, 1.25 ml of a 20% PFA solution were added for 24 h at 37°C to inactivate the bacteria prior to the investigation of mycobacterial lipid profiles (see below).

### Study Population of *Mycobacterium tuberculosis* L1 – East African Indian Strains

Whole genome sequencing datasets for L1 strains were obtained by screening our internal WGS database of 21,000 strains, which have been automatically classified based on the Coll lineage classification system (Coll et al., 2014) for an initial number of 475 genomes. Out of these, low quality datasets (average read coverage ≤ 50 fold and/or coverage breadth ≤ 95%), double sequenced strains and datasets with no metadata were excluded. After a preliminary analysis datasets of low diversity samples originating from possible transmission events with a distance of ≤5 SNPs to at least one other strain were excluded. This resulted in a final dataset of 312 L1 strains from 43 countries.

### Whole Genome Sequencing

For all WGS datasets, libraries were prepared from gDNA using a modified Nextera protocol (Baym et al., 2015) or the Nextera XT kit (Illumina, San Diego, CA, United States) and were sequenced with 2 bp × 100 bp, 2 bp × 150 bp, or 2 bp × 300 bp paired-end reads on an Illumina HiSeq, MiSeq or NextSeq 500 platform as instructed by the manufacturer (Illumina, San Diego, CA, United States).

Raw sequence data (FASTQ files) were analyzed with MTBseq, a semi-automated bioinformatic pipeline for the analysis of MTBC isolates (Kohl et al., 2018). Briefly, reads were mapped to the *M. tuberculosis* H37Rv reference genome (GenBank ID: NC_000962.3) and alignments were refined with regard to base quality re-calibration and alignment corrections for possible PCR artifacts. Further data processing for variant calling and inference of transmission events was done as previously described (Merker et al., 2018). Variants were considered if covered with at least four reads indicating the variant in both forward and reverse read orientation, four reads showing the variant with at least a Phred score of 20, and found with at least 75% allele frequency. For the evaluation of variations in the PDIM and PGL pathway (Siméone et al., 2010), the presence of pathway genes was confirmed for all strains. All datasets reached a coverage depth of at least 50× and a coverage breadth of at least 95% of the reference genome.

### Phylogenetic Inference

After filtering SNPs within a window of 12 bp in the same genome, or located in repetitive regions and resistance-associated genes, the remaining SNP positions from all compared datasets were combined into a concatenated core-SNP alignment. Maximum likelihood (ML) phylogenetic inference was calculated from this alignment only using polymorphic sites by IQ-TREE v1.6.8 (Nguyen et al., 2015), with automatic model selection by ModelFinder Plus (Kalyaanamoorthy et al., 2017) based on the Akaike information criterion (AIC), corrected Akaike information criterion (AICc), and the Bayesian information criterion (BIC) including ascertainment bias correction (ASC), using ultrafast bootstrap approximation (UFBoot) (Hoang et al., 2018) with 1,000 replicates and the -bnni option to account for the possibility of severe model violations. ML trees were midpoint rooted. We detected sub-lineages within the tree based on deep splits with an UFBoot support ≥ 95 and using an hierarchical Bayesian Analysis of Population Structure (hierBAPS) model as implemented in R (RhierBAPS) with a maximum depth of 2 and 20 initial clusters (Cheng et al., 2013). Results were compared to the lineage classification of Coll et al. (2014).

### Sub-Lineage Specific Single Nucleotide Polymorphisms

From the set of detected polymorphic positions, we extracted sets of variants specific for groups of isolates defined either by the phylogenetic analysis or manual inspection, i.e., phylogenetic SNPs characteristic for groups or sub-lineages of the constructed phylogenetic trees. For that, we evaluated the joint list of the polymorphic positions found in the 312 study isolates by filtering the list for SNP alleles specific for each of the targeted groups of isolates. For phylogenetic informative positions for the classification of sub-lineages only SNPs present in 100% of the sub-lineage strains were evaluated. Synonymous SNPs in essential, coding regions were preferred. All strains were reclassified based on the newly selected SNPs.

### Phylogeographic Ancestral State Inference

To infer the geographic origin of the ancestors of L1 lineage we used the information of the patient’s origin defined by the UN-region as discrete states. When strains were isolated in a low incidence TB country (e.g., Europe), the majority of such cases corresponds to immigrants from high incidence countries and it is highly likely that they were infected in their country of origin. Thus, the native geographic range of the strains is the informative criterion for the phylogeographic reconstruction. As no information on the individual living and health, working, travel and immigration history was available, it cannot be excluded that in some cases samples may have been attributed to an incorrect country if infection happened not in the home country. To mitigate an influence on the results, countries were binned into UN-regions. We used the origin information off all 312 strains and the calculated ML-phylogeny for the ancestral reconstruction. First the best model of character evolution was selected by calculating the AIC for an equal-rates (ER) model, a symmetric, un-ordered model (SYM), and an all-rates-different (ARD) model with fitMk from the R package phytools (Revell, 2012) and computing their Akaike weights. Scaled likelihoods for each ancestral state were calculated with the best fitting model ER (aic.w = 0.97) and the ace function from the R package ape (Paradis et al., 2004) using joint maximum likelihood estimation and were mapped back to the ML phylogeny.

### Total Lipid Extracts

Extraction of total lipids from mycobacterial cells was performed following a previously described procedure (Mahrous et al., 2008) with a few modifications. Briefly, the cells were harvested by centrifugation at 1,900 × *g* and 4°C for 10 min. The cell pellet was washed three times with D_2_O (appr. 1 ml per 0.1 g cells), removing the aqueous wash in between by centrifugation (3,700 × *g*, 20°C, 10 min). In a final washing step, the material is transferred with 1–2 ml of D_2_O into a 2 ml Eppendorf safe-lock tube (3,700 × *g*, 20°C, 10 min). After careful removal of the remaining D_2_O and lyophilization for 30 mins, the semi-dry pellet was weighed and subsequently extracted with a 2:1 (v/v) mixture of CDCl_3_ and CD_3_OD (3 ml per milligram of cells). The extraction was performed at 37°C for 90 min with continuous shaking at 250 rpm. After extraction, CD_3_OD (1 ml per milligram of cells) was added to allow the formation of a single solvent phase. The pellet was then separated by centrifugation (3,700 × *g*, 20°C, 10 min). However, in most cases the addition of the double volume of CD_3_OD was necessary to form a single solvent phase. Finally, the supernatant was dried under a stream of nitrogen gas and subsequently freeze-dried and weighted.

### NMR Spectroscopy

Deuterated solvents were purchased from Deutero GmbH (Kastellaun, Germany). NMR spectroscopic measurements were performed in CDCl_3_/CD_3_OD/D_2_O 60:35:8 (v/v/v) at 300 K on a Bruker Avance^III^ 700 MHz (equipped with an inverse 5 mm quadruple-resonance Z-grad cryoprobe). Total lipid extracts were dissolved in a concentration of 6 μg/μl and ^1^H, ^13^C-HSQC NMR lipid profiles were recorded in 3 or 5 mm NMR tubes, respectively, based on the available amount of total lipid extracts. In the case of insufficient amount of lipids, the complete sample was dissolved in 150 μl of solvent mixture and measured using a 3 mm NMR tube. TMS was used as an external standard for calibration of ^1^H (δ_H_ = 0.0 ppm) and ^13^C (δ_C_ = 0.0 ppm) NMR spectra. All data were acquired and processed using Bruker TOPSPIN V 3.0 or higher (Bruker BioSpin Corporation, Billerica, MA, United States).

### Isolation of Glycolipids by Preparative Thin-Layer Chromatography

Total lipid extracts were dissolved in CHCl_3_/CH_3_OH 4:1 (v/v) in a concentration of 20 μg/μl and applied to HPTLC plates (glass-backed 10 cm × 10 cm silica gel 60 F_254_; Merck KGaA, Darmstadt, Germany, 1.05635.0001; up to five mg per plate). Lipids were separated with CHCl_3_/CH_3_OH 95:5 (v/v), and a small sidebar of the plate was stained with Hanessian’s stain (Pirson et al., 2015). Lipid bands (R_f_ range was 0.49–0.54 for **1**, 0.54-0.60 for **2**) were isolated from the silica and recovered by extraction with CHCl_3_/CH_3_OH 2:1 (v/v) for 30 s with thorough vortexing and subsequent centrifugation (1,080 × *g*, 10 min, 4°C; three rounds of collecting and replacing the organic supernatant). After drying under a stream of nitrogen gas, the residual silica was removed by filtration in CHCl_3_/CH_3_OH 2:1 (v/v) using an Acrodisc CR 13 mm syringe filter (0.2 μm PTFE membrane, Pall Corporation, Puerto Rico, United States; washed with 2 ml × 2 ml CHCl_3_/CH_3_OH 2:1 (v/v) prior to use).

### Macrophage Assays

Macrophage infection experiments were performed as described previously (Reiling et al., 2013). In brief, 2 × 10^5^ human monocyte-derived macrophages (hMDMs) were cultured in 500 μl RPMI 1640 (Biochrom, Merck KGaA, Darmstadt, Germany) with 10% FCS and 4 mM L-glutamine (Biochrom, Merck KGaA, Darmstadt, Germany) in 48-well flat-bottom microtiter plates (Nunc, Thermo-Fisher Scientific, Waltham, MA, United States) at 37°C in a humidified atmosphere containing 5% CO_2_. Macrophages were infected with designated MTBC strains with a MOI of 1 bacterium per macrophage (1:1) for 4 h. Subsequently non-phagocytosed bacteria were removed by washing three times with 0.5 ml Hanks’ balanced salt solution (HBSS; Biochrom, Merck KGaA, Darmstadt, Germany) at 37°C. After washing and after 3 days of cultivation, 0.5 ml media was added to the macrophage culture. At day 7 supernatants were completely removed. Macrophage cultures were lysed at 4 h and 7 day post infection by adding 10 μl 10% Saponin solution (Sigma-Aldrich Chemie GmbH, Steinheim, Germany) in HBSS at 37°C for 15 min. Inoculated bacteria and macrophage lysates were serially diluted in sterile water containing 0.05% Tween 80 (Sigma-Aldrich Chemie GmbH, Steinheim, Germany) and plated twice on 7H10 agar containing 0.5% glycerol (Carl Roth, Karlsruhe, Germany) and 10% heat-inactivated Bovine Calf Serum (BioWest, Nuaillé, France). After 3 weeks at 37°C the CFUs were counted. Strain 1797/03 was used as an internal control in all experiments performed.

## Results

### *Mycobacterium tuberculosis* Complex Lineage 1 Phylogeny

**FIGURE 2:**
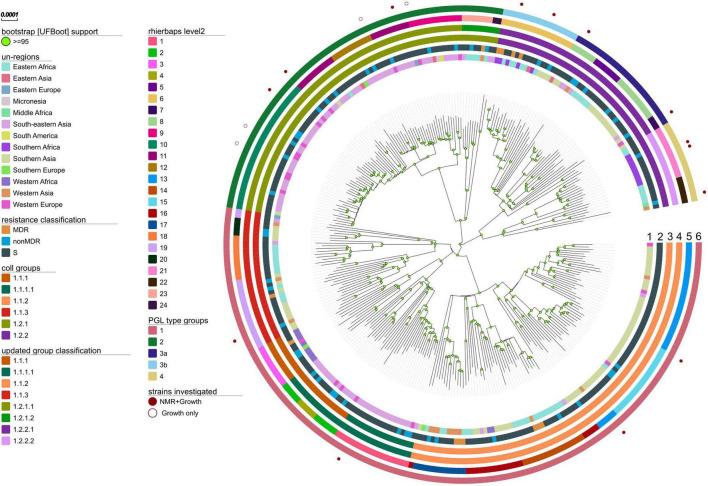
Phylogeny, sub-lineages and PGL type groups of L1 MTBC strains. Maximum likelihood phylogeny (substitution model TVM + F + ASC as automatically determined by ModelFinder Plus of IQ-TREE, 1000 bootstrap replicates (UFBoot support ≥ 95 marked with green circles on nodes), built from the concatenated SNP alignment) of 312 MTBC L1 strains spanning 13 UN-regions. Tracks from the inside to outside show color coded for each strain: *circle 1* – sample origin defined as patients country of birth; *circle 2* – the antibiotic resistance classification in susceptible, not MDR and MDR strains (no preXDR or XDR were found); *circle 3* – the MTBC sub-lineage classification by Coll et al. (2014); *circle 4* – the here redefined sub-lineage classification; *circle 5* – the nested population structure calculated with RhierBAPS by hierarchically clustering the DNA sequence data on the second level; *circle 6* – defined PGL type groups based on the by branching, common mutation patterns and observed PGL types; strains analyzed with NMR for their PGL types and growth in macrophages (filled red circles) or only for growth (red circles) are indicated.

To analyze the global population structure of L1 strains we compiled a WGS dataset of 312 L1 strains from 43 countries, spanning 13 UN-regions that comprise all major geographical areas with reported occurrence of L1 strains (Figure 2, Supplementary Figure 1, and Supplementary Table 1). Sub-lineages have been automatically assigned by using the currently most widely used classification system of Coll et al. (2014). Our WGS data analysis revealed 35,517 single nucleotide polymorphisms (SNPs) occurring in the genome of at least one strain in comparison to the reference genome of *M. tuberculosis* H37Rv. All SNP positions were combined into a concatenated sequence alignment and used for downstream data analysis, e.g., phylogenetic tree calculations, definitions of sub-lineages and their signature SNPs. The Maximum likelihood (ML)-phylogeny revealed a diverse population structure with an overall number of eight sub-lineages, out of which six have been described by Coll et al. (2014) (Figure 2 and Supplementary Figure 2). For our high-resolution analysis, we updated the sub-lineage classification based on Coll et al.: Coll lineage 1.2.2 is split into two sub-lineages named 1.2.2.1 and 1.2.2.2, and Coll lineage 1.2.1 is split into 1.2.1.1 and 1.2.1.2. Additionally, we redefined Coll groups 1.1.1 and 1.1.1.1 to reflect a deeper split in the phylogeny. For all sub-lineages, we assigned new signature SNPs that allow rapid L1 sub-lineage classification (Supplementary Table 2).

**FIGURE 3:**
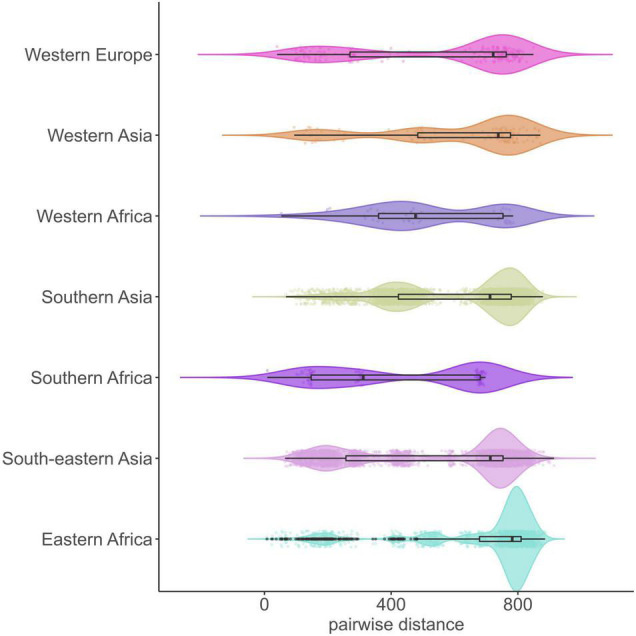
Genetic diversity of 312 L1 MTBC strains within UN-regions. Violin plots show the pairwise SNP distance of strains for each geographic region. Boxplots within the violin represent the 25th and 75th percentile, black horizontal line the median, black dots are outliers and colored dots the individual pairwise SNP distance values (jitter = 0.1). UN-regions with a total strain count smaller than 10 are not shown.

**FIGURE 4:**
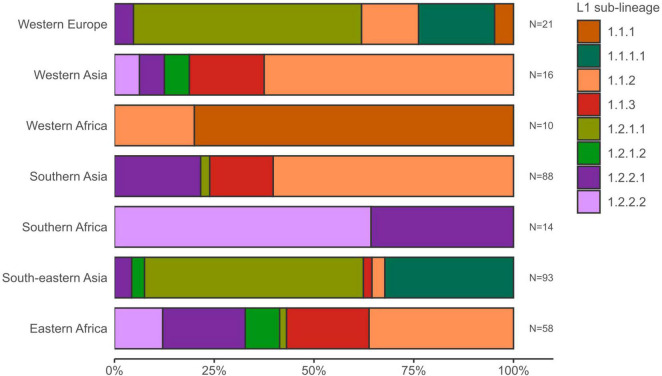
Sub-lineage diversity of L1 MTBC 1 strains within UN-regions. Stacked bars show for each geographic region the sub-lineage presence and proportions of the L1 strain population. UN-regions with a total strain count smaller than 10 are not shown.

The average pairwise distances (avg. pwd) of distinct SNP positions in the sub-lineages ranged between 205 (median = 199) SNPs for group 1.2.1.1 to 556 (median = 642) SNPs for 1.1.3 (Supplementary Figure 3). On the geographic level, the region with the lowest avg. pwd distance of 437 (median = 312) was Southern Africa followed by Western Africa (avg. pwd = 518, median = 477) (Figure 3). In both regions only two sub-lineages were identified, 1.2.2.1 and 1.2.2.2 in Southern Africa and 1.1.1 and 1.1.2 in Western Africa, respectively (Figure 4). Sub-lineage 1.1.1 of this classification is, apart from two strains from Europe, exclusively found in Western Africa supporting this sub-lineage redefinition. The highest L1 population diversity was found among strains from Eastern Africa (avg. pwd = 694, median = 782) with six L1 sub-lineages present. Strains of six sub-lineages were also found to be present in South-eastern Asia (avg. pwd = 544, median = 713), but in contrast to Eastern Africa two sub-linages (1.1.1.1 and 1.2.1.1) were dominant, which were nearly exclusively found in this region.

**FIGURE 5:**
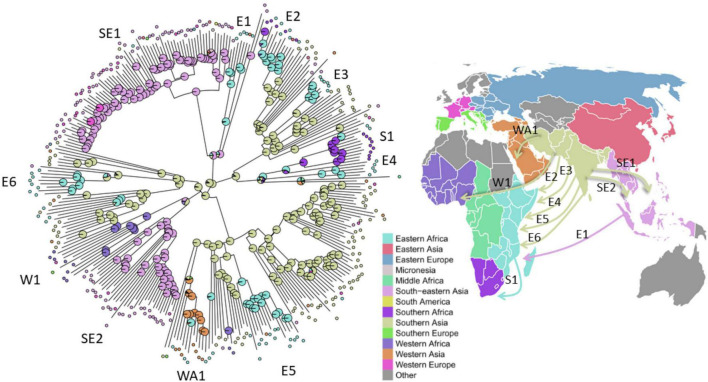
Inference of the L1 strains geographic origin and migration. *Left*: Scaled likelihoods of geographic ancestral states as calculated by joint maximum likelihood estimation mapped onto nodes of the maximum likelihood phylogeny of the 312 L1 strains, colored according to the UN-region (pie charts). Selected migration events of L1 strains to South-eastern Asia (SE), to Eastern Africa (E), to Southern Africa (S), to Western Africa (W), and to Western Asia (WA) are numbered and marked on the tree. *Right*: Directional global movement of the selected multiple migration events of L1 strains.

To better understand the global spread and origin of L1 strains, we mapped the geographical regions the strains have been obtained from on the ML-phylogeny and performed a phylogeographic ancestral state reconstruction analysis (Figure 5). The ancestral origin of L1 strains was predicted to be Southern Asia (89% scaled likelihood), from which the dispersal of strains of particular L1 sub-lineages into other geographical regions likely occurred. For example, our analysis indicates six separate and independent introductions of strains of distinct L1 sub-lineages into Eastern Africa by human migration (Figure 5). From Eastern Africa, strains of particular L1 sub-lineages were then further distributed to Southern Africa (S1, Figure 5). Furthermore, L1 strains were likely introduced from Southern Asia to Western Asia (WA1, Figure 5) and Western Africa (W1, Figure 5) at least once and different sub-lineages twice to South-eastern Asia (SE1-2, Figure 5).

Phenolic glycolipids represent polyketide-derived MTBC virulence factors that require a complex biosynthesis, therefore we screened the WGS data for variants in genes involved in this pathway. Overall, we found 46 variations with 29 non-synonymous mutations also comprising the previously reported Q294Stop in Rv2962c (Krishnan et al., 2011) or a 1 bp insertion in Rv2946c (Supplementary Table 3).

### 2D ^1^H,^13^C-Heteronuclear Single Quantum Coherence NMR Lipid Profiling

**FIGURE 6:**
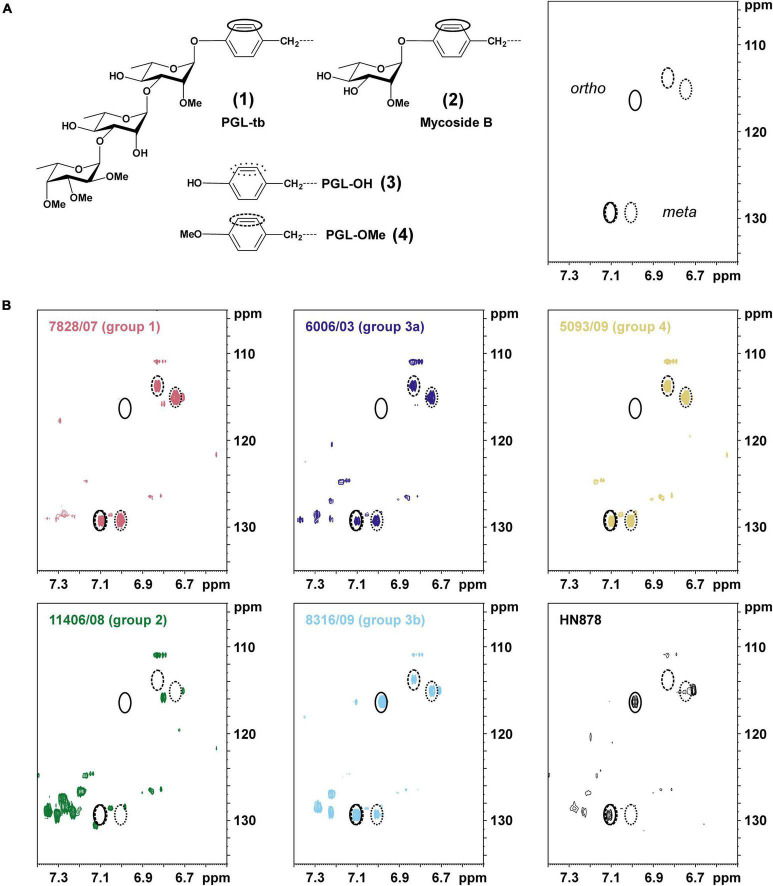
Specific signal pattern in ^1^H,^13^C-HSQC NMR maps indicate the presence or absence of phenolphthiocerol dimycoserosates and/or phenolic glycolipids (PGLs; mycoside B/PGL-tb) in total lipid extracts of selected *M. tuberculosis* L1 clinical isolates. **(A)** Chemical structures of the (glyco-)phenol part of phenolic glycolipids (**1,2**) and/or phenolphthiocerol dimycoserosates (**3,4**), for full structure see Figure 1. In the right part of this panel, the expected signal pattern for the phenol *ortho*- and *meta*-position of molecules **1–4** in the respective section (δ_H_ 7.40–6.50 ppm; δ_C_ 135–105 ppm) of a ^1^H,^13^C-HSQC NMR map are shown. Circles with solid lines represent these positions for a glycosylated phenolic ring (as present in **1,2**), dotted lines for PGL-OH (**3**) and dashed lines for PGL-OMe (**4**), respectively. **(B)** The specific chemical shift region of the ^1^H,^13^C-HSQC NMR maps of the total lipid extracts are depicted for representative strains of each L1 sub-lineage as well as L2 strain Beijing HN878 as a PGL-tb containing strain. The observed C,H-correlations enable a specific determination of the present PGL types (Mahrous et al., 2008) in all studied clinical isolates (Table 2).

In the next step of our study, 14 strains of different L1 sub-lineages were investigated to determine the type of synthesized PGLs using 2D ^1^H,^13^C-heteronuclear single quantum coherence (HSQC) NMR (Figure 6). Strain HN878 (Beijing) was used as a reference for a PGL-tb [**(1)**; Figure 1] producing strain (Mahrous et al., 2008, 2009). The chemical shift region specific for the aromatic C–H-correlations is depicted for representative strains of each sub-lineage in Figure 6, for all other analyzed strains in Supplementary Figure 4.

**TABLE 1:** ^1^H (700.4 MHz) and ^13^C NMR (176.1 MHz) chemical shift data (δ, ppm) [*J*, Hz] for PGL-tb **(1)** isolated from *Mycobacterium canettii* strain 3040/99, recorded in CDCl_3_/CD_3_OD/D_2_O 60:35:8 (v/v/v) at 300 K.

Residue (assignment)	H-1 *C-1*	H-2 *C-2*	H-3 *C-3*	H-4 *C-4*	H-5 *C-5*	H-6 *C-6*
2,3,4-tri-*O*-methyl-α-Fuc*p*-(1→ [**Fuc**]	5.25 [d, 3.8] *99.2*	3.62–3.58* *78.4*	3.71-3.67* *80.1*	3.56–3.53* *79.3*	4.19–4.14* *67.2*	1.25 [d, 6.6] *16.3*
OCH_3_		3.54 [s] *58.8*	3.52 [s] *57.7*	3.59 [s] *61.8*		
→3)-α-Rha*p*-(1→ [**Rha^II^**]	5.05 [d, 1.2] *102.9*	4.10–4.07* *70.8*	3.82–3.78* *80.6*	3.66–3.60* *71.5*	3.87–3.83* *69.4*	1.35 [d, 6.3] *17.8*
3)-2-*O*-methyl-α-Rha*p*-(1→ [**Rha^I^**]	5.49 [d, 1.2] *95.4*	3.74–3.71* *80.6*	4.03–4.00* *78.9*	3.58–3.53* *72.0*	3.76–3.71* *69.6*	1.29–1.25* *17.5*
OCH_3_		3.53 [s] *58.9*				

	**OC**	***ortho* (g^#^)**	***meta* (h^#^)**	***para***		
→O-phenol	*154.7*	6.99 [d, 8.5] *116.4*	7.11 [d, 8.5] *129.6*	*71.2*		

**Further indicative groups^#^**						
a		4.88–4.83* *70.8*				
b		3.35 [s] *57.5*				
c		2.94–2.90* *87.3*				
d		2.57–2.52* *38.1*				
f		1.17 [d, 7.0] *18.6*				
CH_2_ (@ *para*-position of the phenolic ring)		2.58–2.54* *35.4*				
a – *CH*_2_ – a		1.82–1.76* *38.7*				
a – *CH*_2_ – CH_2_		1.61–1.56*, 1.56–1.48* *34.8*				

^#^Following the nomenclature of Pérez et al. (2004) (see Figure 1). *Non-resolved multiplet.

**TABLE 2:** Summary of the observed phenolic glycolipids (PGL) types in the 14 selected clinical L1 MTBC strains in correlation to the respective causative variations in PGL biosynthesis genes.

*Mtb* strain	L1 sub-lineage^a^	PGL group^a^	PGL profile^b^	Genetic variation in PGL biosynthesis associated genes^c^
1797/03	1.1.2	1	PGL-OH, PGL-OMe	Rv2962c Gln294Stop
7828/07	1.1.2	1	PGL-OH, PGL-OMe	Rv2962c Gln294Stop
9870/09	1.1.1.1	1	PGL-OH, PGL-OMe	Rv2962c Gln294Stop
947/01	1.1.3	1	PGL-OH, PGL-OMe	Rv2962c Gln294Stop
4858/08	1.2.1.1	2	No	1 bp insertion in Rv2946c
11406/08	1.2.1.1	2	No	1 bp insertion in Rv2946c
5325/09	1.2.1.1	2	No	1 bp insertion in Rv2946c
8316/09	1.2.2.1	3b	Mycoside B, PGL-OH, PGL-OMe	Rv2958c Tyr182Asp, Rv2962c Leu46Gly
5239/09	1.2.2.1	3b	Mycoside B, PGL-OH, PGL-OMe	Rv2958c Tyr182Asp, Rv2962c Leu46Gly
6006/03	1.2.2.1	3a	PGL-OH, PGL-OMe	Rv2962c Leu46Arg
682/08	1.2.2.1	3a	PGL-OH, PGL-OMe	Rv2962c Leu46Arg
5093/09	1.2.2.2	4	PGL-OH, PGL-OMe	1 bp insertion in Rv2962c
4300/09	1.2.2.2	4	PGL-OH, PGL-OMe	1 bp insertion in Rv2962c
8057/11	1.2.2.2	4	PGL-OH, PGL-OMe	1 bp insertion in Rv2962c

*^a^According to Figure 2; ^b^For chemical structures see Figures 1, 6A; ^c^Position of amino acid exchange after start codon.*

The NMR spectrum of strain HN878 (Figure 6) contains the two signals specific for a glycosylated PGL (closed circles), here PGL-tb (**1**). Consistent with their specific mutation patterns (Supplementary Table 3), all strains of sub-lineages 1.1.1, 1.1.1.1, 1.1.2, 1.1.3 (PGL group 1, red), and 1.2.2.2 (PGL group 4, yellow) according to our updated classification showed signals in their respective NMR lipid profile indicating the presence of PGL-OH [**(3)**; dotted circles] and PGL-OMe [**(4)**; dashed circles]. Strains of sub-lineages 1.2.1.1 and 1.2.1.2 (PGL group 2, green), sharing a 1 bp insertion in Rv2946c, exhibit none of these signals. Very interestingly, some spectra of strains of sub-lineage 1.2.2.1 contain a glycosylated PGL (PGL group 3b, light blue) in addition to PGL-OH and PGL-OMe, which are the molecules present in the spectra of the other members of this sub-lineage (PGL group 3a, dark blue). To the best of our knowledge, these are the first examples of *M. tuberculosis* strains not belonging to L2 producing a glycosylated PGL. However, the shifts of the aromatic C-H-correlations are not sufficient to determine the number of bound glycosyl residues.

**FIGURE 7:**
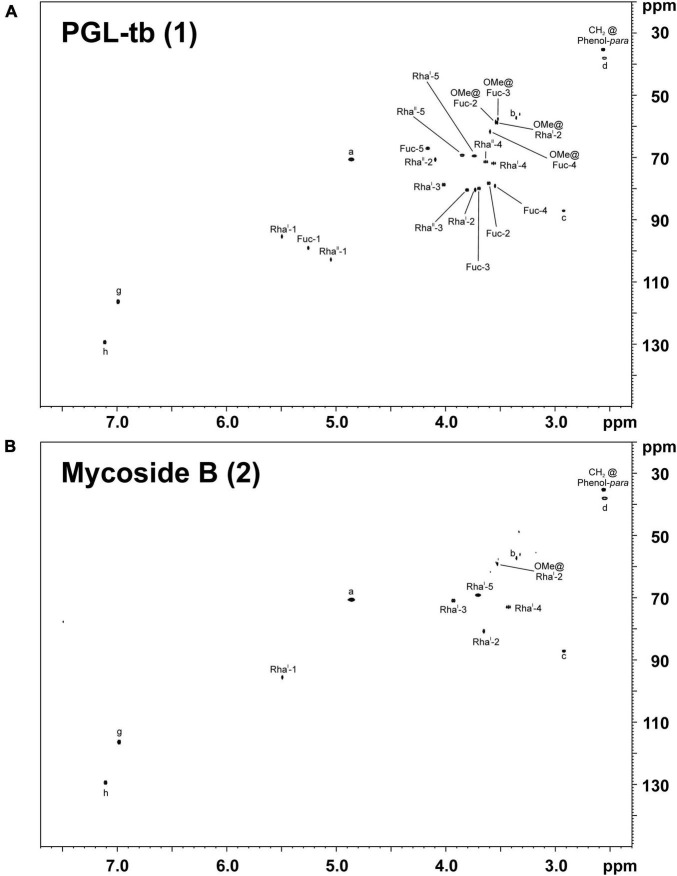
^1^H,^13^C-HSQC NMR spectra of PGLs isolated from the total lipid extract of *M. canettii* strain 3040/99 grown in ^13^C-enriched medium. The chemical shift regions of δ_H_ 7.70–2.30 ppm and δ_C_ 150–20 ppm are depicted for **(A)** PGL-tb (**1**) and **(B)** mycoside B (**2**). For the respective chemical structures and nomenclature see Figure 1.

**FIGURE 8:**
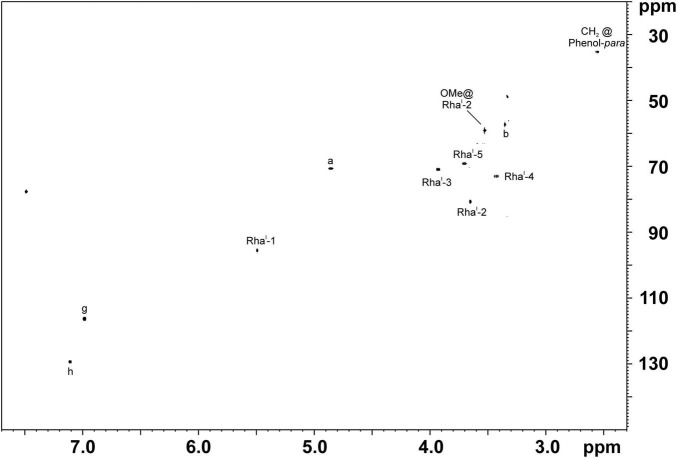
^1^H,^13^C-HSQC NMR spectra of PGLs isolated out of the total lipid extract of L1 strain 8316/09 grown in ^13^C-enriched medium. The chemical shift region of δ_H_ 7.70–2.30 ppm and δ_C_ 150–20 ppm is depicted. Comparison with spectra shown in Figure 7 clearly proofs the presence of mycoside B (**2**) in this strain. For the respective chemical structure and nomenclature see Figure 1.

Therefore, we isolated PGL-tb (**1**) and mycoside B (**2**) from an *Mycobacterium canettii* clinical isolate (3040/99) as reference molecules for the investigation of the PGL from L1 strain 8316/09 (sub-lineage 1.2.2.1). *Mycobacterium canettii* was chosen for PGL-tb (**1**) and mycoside B (**2**) isolation, since *M. canettii* is the only member of the MTBC known to produce both molecules at the same time in sufficient amounts. Mycoside B (**2**), the major PGL present in *M. bovis*, has been shown to be identical to that of *M. canettii* (Daffé et al., 1988). The structure of PGL-tb (**1**) isolated from *M. canettii* (Daffé et al., 1987) and from *M. tuberculosis* L2 strains (Huet et al., 2009) is almost identical showing equality in the glycosidic part. The reference molecules showed the indicative three [PGL-tb, **(1)**] or one [mycoside B, **(2)**] C-H-correlations for anomeric protons in the HSQC spectra, respectively (Figure 7 and Table 1). The spectrum for the PGL isolated from L1 strain 8316/09 showed the presence of mycoside B (**2**) in this strain (Figure 8) if compared to the reference, suggesting in turn that the SNP in Rv2958c (resulting in Y182D on the amino acid level) might render the glycosyltransferase inactive. The observed correlation between SNP analysis and PGL structures led us to the definition of five “PGL type groups”. Remarkably, sub-lineage 1.2.2.1 comprises PGL type groups 3a and 3b, respectively (Figure 2). The observed PGL types are summarized in association to the crucial genetic variants in PGL biosynthesis associated genes in Table 2.

### Growth of L1 Strains in Human Macrophages

**FIGURE 9:**
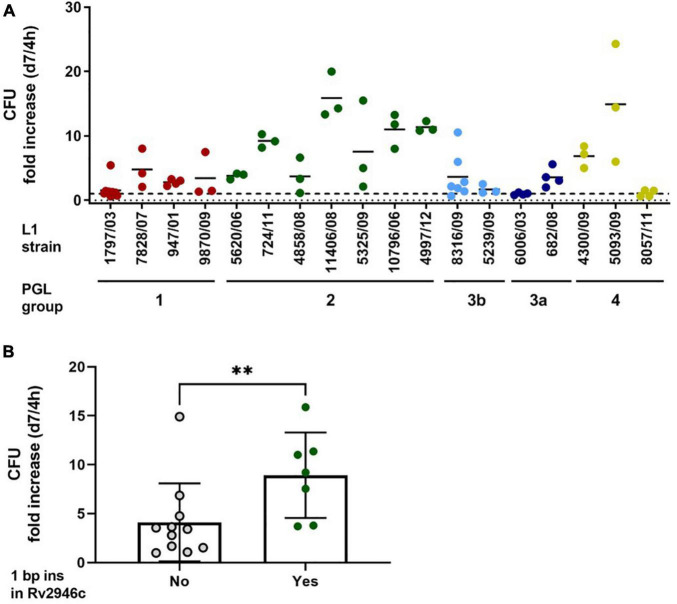
Growth analysis of L1 MTBC strains in human macrophages. **(A)** Human monocyte-derived macrophages (hMDMs) were infected with the indicated strains with an MOI of 1:1 for 4 h and 7 days. Quantification of viable CFU at 4 h and 7 days post infection was conducted by lysis of monolayers, serial dilution, and plating on 7H10 medium from at least three independent experiments with cells from different donors. Data shown represent a summary of ten experiments performed with cells generated from independent healthy donors with each L1 isolate tested at least three times (*n* = 3 or 4). Strain 8316/09 was used in more experiments (*n* = 7) for internal control purposes, as it represents the PGL group 3b strains characterized by mycoside B (**2**) production. Strain 1797/03 (*n* = 10) was used as an internal control in all experiments performed. **(B)** Fold growth of L1 MTBC strains is dependent on the presence of the 1 bp insertion in Rv2946c (yes: PGL group 2; no: all other PGL groups). Shown are the means ± SEM of strains analyzed in panel **(A)** (^**^P = 0.0083, Mann-Whitney *U* test).

Next, we performed infection experiments in human monocyte-derived macrophages to assess the growth behavior of the analyzed L1 strains in the primary host cell of MTBC strains. Cells were infected with a multiplicity of infection (MOI) of 1:1 for 4 h, subsequently washed and analyzed for the cellular bacterial burden on day 7 post infection, as previously described (Reiling et al., 2013). The L1 strains 1797/03 and 947/01 as well as two other strains belonging to the same genetic group (sub-lineages 1.1.1, 1.1.1.1, 1.1.2, and 1.1.3; PGL group 1, red), showed a limited growth behavior (reduced to persistent growth). This ranged from a fold increase of 1.5 (1797/03) to 4.7 (7828/07) if the CFU after 7 days was related to bacterial numbers in the macrophage culture after 4 h (Figure 9A). Similar observations were made for the four strains of sub-lineage 1.2.2.1 (PGL group 3a/b, blue) and the three strains analyzed for sub-lineage 1.2.2.2 (PGL group 4, yellow). While strains of these three groups revealed a comparable and rather similar growth behavior, the L1 strains belonging to sub-lineage 1.2.1.1. (PGL group 2, green), which do not express PGLs and phenolphthiocerol dimycoserosates, showed a more diverse pattern. Two isolates (5620/06, 4858/06) matched the growth characteristics of the strains of the other groups, however, five strains showed a significantly increased growth capacity in human macrophages. The fold increase rates ranged from 9.1 (724/11) to 15.8 (11406/08). A comparative analysis of these strains carrying a 1 bp insertion in Rv2946c to all other strains analyzed showed a significantly enhanced average growth rate (*P* = 0.0083; Figure 9B). The differences in macrophage growth characteristics were not due to reduced or increased uptake of the bacteria after 4 h, since the majority of L1 strains added to the cells in the respective experiment (except 11406/08) were taken up by the macrophages in a comparable range (6.3 ± 2.3%; mean ± SD) (Supplementary Figure 5).

## Discussion

Our study presents a detailed analysis of the phylogeography for L1 strains and correlates the observed genetic diversity with differences in the PGL biosynthesis. These lipids have been shown to be important immunomodulators and their differential production may indeed influence host-pathogen interaction and the outcome of TB infection. Phylogenetic analysis of 312 L1 strains from 43 countries representing 13 UN regions confirmed a highly diverse phylogeographical population structure that is associated with a specific PGL biosynthesis. Indeed, testing the growth behavior in a macrophage infection model indicates that the different PGL profiles may influence the host-pathogen interaction. Ancestry analysis indicated that L1 strains likely originated from Southern Asia, which is in line with previous studies (Couvin et al., 2019; Menardo et al., 2021). Indeed, the data obtained here are in accordance with a previous study suggesting an “out of India” scenario for L1 stains with subsequent spread to Eastern Africa and further diversification within Africa (O’Neill et al., 2019).

Based on our phylogenetic analysis, we redefined the L1 sub-lineage classification that can now be linked to distinct geographical occurrence and pathobiology, e.g., variations in PGL profiles. We provide signature SNPs for the redefined L1 sub-lineages, thus allowing an easy to perform L1 strains sub-lineage classification in future studies. In particular, Coll sub-lineage 1.2.1 is split in 1.2.1.1, mainly present in South eastern Asia, and 1.2.1.2 mainly occurring in Western Asia and Eastern Africa. Sub-lineage 1.2.2 is split into two sub-lineages named 1.2.2.1 and 1.2.2.2. Noteworthy, an update to the Coll-nomenclature (Coll et al., 2014) was provided by Napier et al. (2020), recently. However, as in that work sub-lineage names were severely shifted, we here decided to build on the established classification scheme from Coll et al. (2014) for reasons of clarity. Nonetheless, the correlation between sub-lineages defined in our study and both other nomenclature systems (Coll et al., 2014; Napier et al., 2020) is depicted in Supplementary Figure 2. Indeed, the presence of multiple sub-lineages within the L1 lineage as shown in our work is in agreement with Napier et al. (2020). Even more, based on the RhierBAPS classification (level 2) performed in this study, a total of 24 L1 sub-lineages are conceivable. These sub-lineages comprise all sub-lineages observed by Napier et al. (2020) and suggest additional ones. Still, we kept the classification in eight sub-lineages as they capture the main subtypes and PGL biosynthesis prototypes.

Interestingly, linking L1 subtypes with geographical occurrence, it became obvious that strains of the two sub-lineages 1.2.2.1 and 1.2.2.2 were identified with the highest per country ratio in Southern Africa, suggesting an adaptation of the pathogen to a specific host population. This is supported by the fact that strains in Southern Africa showed the lowest average pairwise distance of 437 among all regions. However, further data are needed to investigate potential patho-adaptation of strains of particular L1 sub-lineages.

One unique feature of our study is that we could correlate distinct PGL type groups to particular sub-lineages. Lipid compositions differ among mycobacterial species and many of these lipids have been shown or are at least suspected to interfere with the host immune system (Jackson, 2014), among them PGL and PDIM (Forrellad et al., 2013). A first indication of immune modulation or even “super virulence” induction for MTBC strains was obtained by investigation of a subset of clinical strains from the L2 lineage that displayed PGL-tb (**1**; Figure 1; Reed et al., 2004). Further studies showed that this lipid is capable of modulating the early host cytokine response (Stadthagen et al., 2006), and contributes to the manipulation of macrophage recruitment (Cambier et al., 2014), but does not confer hypervirulence itself (Sinsimer et al., 2008). Remarkably, all MTBC strains of the globally distributed highly successful L4 do not produce PGLs due to a fixed 7 bp deletion in *pks15/1* compared to L2 strains, leading to a complete termination of phenolphthiocerol dimycoserosate biosynthesis (Constant et al., 2002). Accordingly, different PGL production patterns may rather represent a host-pathogen adaptation mechanism than an overall virulence marker.

With this regard, our data confirm that L1 sub-lineage specific PGL types are highly interesting considering the differences in the geographical spread of strains from different L1 sub-lineages and macrophage growth profiles. Remarkably, all investigated L1 strains have mutations in this particular biosynthesis pathway. This suggests that the alteration of PGLs is potentially important in the evolution of L1 strains. The majority of strains contained PGL-OMe (**3**) and PGL-OH (**4**) due to a stop-codon (Q294Stop; sub-lineages 1.1.1, 1.1.1.1, 1.1.2, and 1.1.3) or a 1 bp insertion in Rv2962c (sub-lineage 1.2.2.2), respectively. Interestingly, strains belonging to sub-lineages 1.2.1.1 and 1.2.1.2 showed a 1 bp insertion in Rv2946c (*pks1*), leading to a complete abolishment of phenolphthiocerol dimycoserosate biosynthesis. Even more intriguing are the observed Rv2962c SNPs in strains of sub-lineage 1.2.2.1 and the resulting PGL profiles (groups 3a and 3b). While group 3a strains only produce PGL-OMe (**3**) and PGL-OH (**4**), group 3b strains contain mycoside B (**2**) suggesting a restoration of the biosynthetic pathway by the second mutation. This is most likely also influenced by a further group 3b specific SNP in Rv2958c (Tyr182Asp), the next acting glycosyltransferase in PGL biosynthesis (Pérez et al., 2004), which is neither present in group 3a nor in the other groups.

Mycoside B has been described so far in strains of other species of the MTBC like *M. africanum*, *M. pinnepedii*, *M. bovis*, and *M. microti*, but not in *M. tuberculosis* strains. In these species, the occurrence of this PGL type was linked to a frameshift mutation in Rv2958c orthologs (Malaga et al., 2008). Further studies are needed to biochemically validate these assumptions by targeted genetic modifications to substantiate the functional impact of the described SNPs, but a link seems quite likely. In summary, our data give a first insight into PGL diversity and their potential role in host-specific adaptation of L1 strains.

The uptake and the growth characteristics of the analyzed L1 strains in human primary macrophages differs between individual strains. Overall, the investigated L1 strains showed limited to moderate growth in macrophages, which corroborated previous observations and was also mirrored in *in vivo* experiments using aerosol infected mice (Reiling et al., 2013). However, strains belonging to sub-lineages 1.2.1.1 and 1.2.1.2 (PGL group 2) with a 1 bp insertion in Rv2946c (*pks1*), leading to a complete termination of phenolphthiocerol dimycoserosate biosynthesis, which is also a characteristic of L4 strains (Constant et al., 2002), showed in average a more prominent growth in macrophages compared to the average growth of all other analyzed L1 strains (*P* = 0.0083; Figure 9B). These data suggest that either full PGL synthesis (PGL-tb), e.g., found in some L2 strains (Reed et al., 2004, Reed et al., 2007), or complete loss of PGL as observed in L4 strains (Constant et al., 2002) and in most L2 strains (Sinsimer et al., 2008) as well as shown here for the newly defined L1 sub-lineage 1.2.1.1 (Figure 2) may represent, among other factors, an advantage for MTBC strain replication in the host. Notably, strains that are unable to produce PGL-tb (**1**) due to a frameshift in *pks15/1* are still able to synthesize pHBAD, the glycosidic precursor building blocks for PGL synthesis (Pérez et al., 2004). Since these molecules can suppress the innate immune response of the infected host like the PGL themselves (Stadthagen et al., 2006; Bourke et al., 2014). Thus, their presence is likely favorable for the mycobacterial pathogen. It was shown that deletion of glycosyl transferase Rv2962c leads to the termination of PGL and also pHBAD synthesis (Pérez et al., 2004). Thus, strains with a non-functional glycosyl transferase Rv2962c, comprising all L1 strains investigated here except strains of sub-lineages 1.2.1.1 and 1.2.1.2, are not capable of synthesizing such molecules. Only the population of sub-lineages 1.2.2.1 that belongs to PGL group 3b has at least a partially restored function. Nonetheless, these strains still synthesize the phenolic PDIM PGL-OH (**3**) and PGL-OMe (**4**), which are present in the other members of this sub-lineage belonging to PGL group 3a as well as sub-lineages 1.1.1, 1.1.1.1, 1.1.2, 1.1.3, and 1.2.2.2. However, the specific impact of PGL-OH (**3**) and PGL-OMe (**4**) on MTBC infection is to the best of our knowledge elusive to date.

Taken together, our study emphasizes a high population diversity of L1 strains that correlates to a distinct phylogeographical population structure and associated to distinct sub-lineage specific PGL profiles. Our finding that strains within a given sub-lineage can vary in their synthesized PGL type highlights their importance for host-pathogen coevolution even at the sub-lineage level. In this regard, the current study underscores the importance of genomic diversity of MTBC strains for their pathobiology. Further in-depth analyses, e.g., using cells from donor populations where L1 strains are most abundant, would be of great interest for studies focusing on the function of these molecules. In addition, the use of more elaborated models is urgently needed to decipher the virulence characteristics of selected L1 individual strains to further prove this concept.

## Data Availability Statement

The datasets presented in this study can be found in online repositories, their accession numbers are listed in Supplementary Table 1.

## Ethics Statement

The studies involving human participants were reviewed and approved by Ethics Committee of the University of Lübeck (14-032). The participants provided their written informed consent to participate in this study.

## Author Contributions

NG, SN, NR, and SH designed and guided the study. NG, CU, SN, NR, and SH wrote the initial manuscript. NG, CU, and NR produced the figures. RD, UG, SG, YS, NN, CK, SV, GK, SA, SA-H, PN, AR, MH, and FM collected the mycobacterial isolates. CU, TK, and SH performed the WGS experiments. CU and TK performed the bioinformatics analysis. NG, LG, KD, DH, and US conceived and performed lipid isolations. NG and LG performed and analyzed NMR experiments. NR and SH conceived and supervised the microbiology culturing. NR conceived and supervised macrophage experiments. NG, CU, LG, SM, DS, SN, NR, and SH contributed the ideas and interpreted the results. All authors read and approved the manuscript.

## Conflict of Interest

The authors declare that the research was conducted in the absence of any commercial or financial relationships that could be construed as a potential conflict of interest.

## Publisher’s Note

All claims expressed in this article are solely those of the authors and do not necessarily represent those of their affiliated organizations, or those of the publisher, the editors and the reviewers. Any product that may be evaluated in this article, or claim that may be made by its manufacturer, is not guaranteed or endorsed by the publisher.
