# Supplementary material for: Sub-Lineage Specific Phenolic Glycolipid Patterns in the Mycobacterium tuberculosis Complex Lineage 1
Source: Front Microbiol. 2022 Mar 8;13:832054. doi: 10.3389/fmicb.2022.832054 (PMC8957993; doi:10.3389/fmicb.2022.832054)
Supplement: Supplementary file 4 [file Data_Sheet_1.pdf]

Supplementary Material

Sub-lineage specific phenolic glycolipid patterns in the *Mycobacterium tuberculosis* complex Lineage 1

Nicolas Gisch[1*‡], Christian Utpatel[2‡], Lisa M. Gronbach[1], Thomas A. Kohl[2], Ursula Schombel[1], Sven Malm[2†], Karen M. Dobos[3], Danny C. Hesser[3], Roland Diel[4], Udo Götsch[5], Silke Gerdes[6], Yassir A. Shuaib[7,8], Nyanda E. Ntinginya[9], Celso Khosa[10], Sofia Viegas[10], Glennah Kerubo[11], Solomon Ali[12], Sahal A. Al-Hajoj[13], Perpetual W. Ndung'u[14], Andrea Rachow[15,16], Michael Hoelscher[15,16], Florian P. Maurer[17,18], Dominik Schwudke[1,19,20], Stefan Niemann[2,19*§], Norbert Reiling[19,21§] & Susanne Homolka[2§]

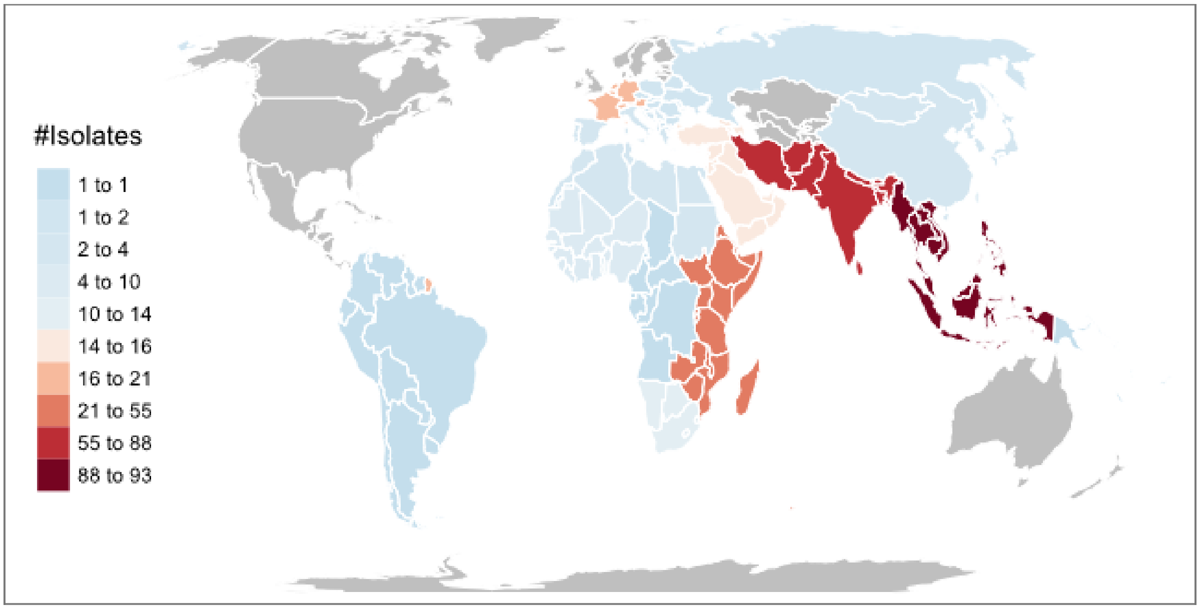

Supplementary Figure 1. L1 strain count distribution for the UN geographic region. Increasing strain count is depicted from blue to red for each region and categories were defined with the Jenks natural breaks classification method for best arrangement of values into different classes.

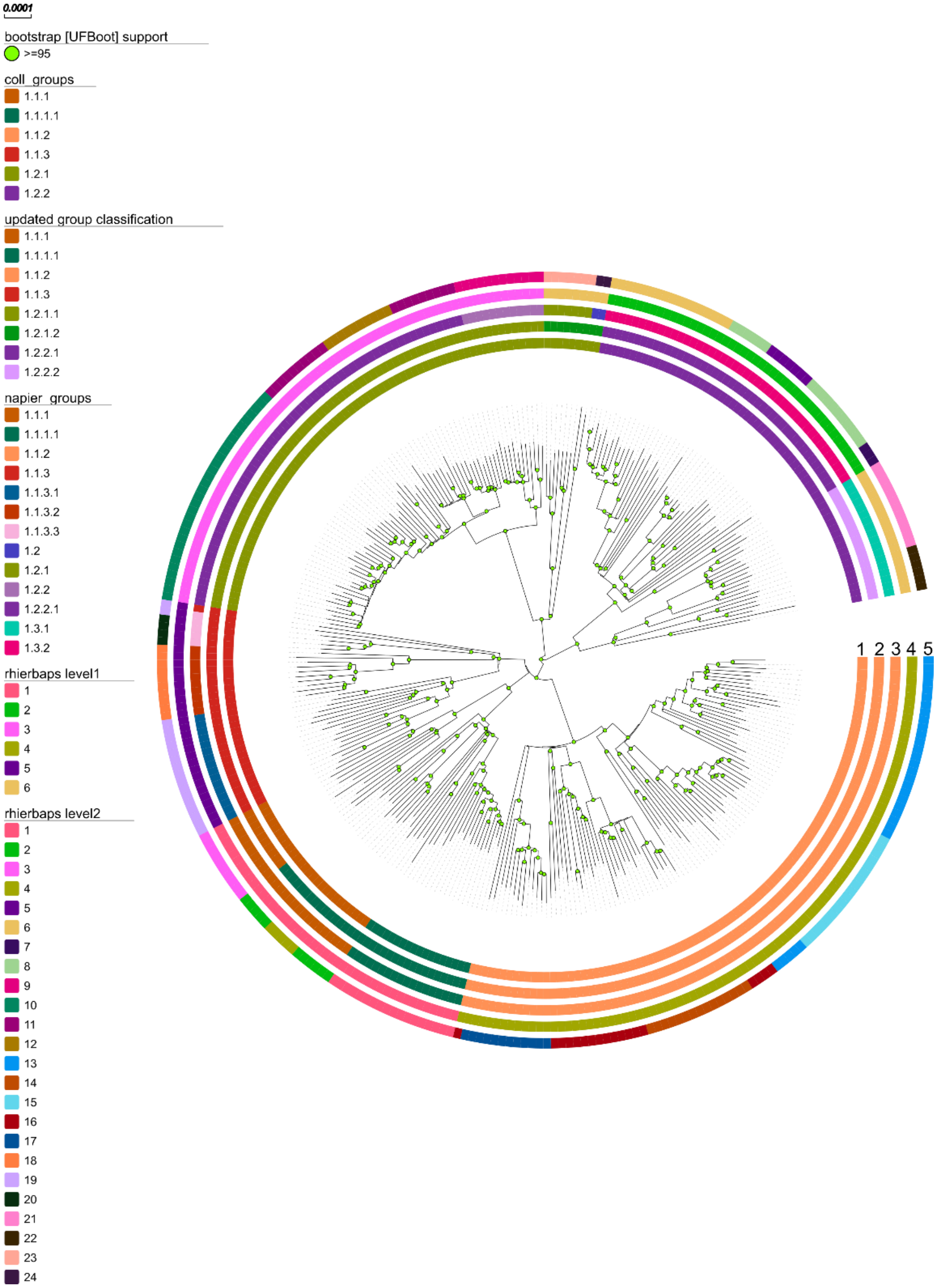

Supplementary Figure 2. Sub-lineage classification of MTBC L1 strains. Maximum likelihood phylogeny (substitution model TVM+F+ASC as automatically determined by ModelFinder Plus of IQ-TREE, 1000 bootstrap replicates (UFBoot support >=95 marked with green circles on nodes), built from the concatenated SNP alignment) of 312 MTBC L1 strains spanning 13 UN-regions. Tracks from the inside to outside show color coded for each strain: *circle 1* - the MTBC sub-lineage classification by Coll et al.[1]; *circle 2* - the here redefined sub-lineage classification; *circle 3* - the updated classification by Napier et al.[2]; the nested population structure calculated with RhierBAPS by hierarchically clustering the DNA sequence data on the first (*circle 4*) and second (*circle 5*) level.

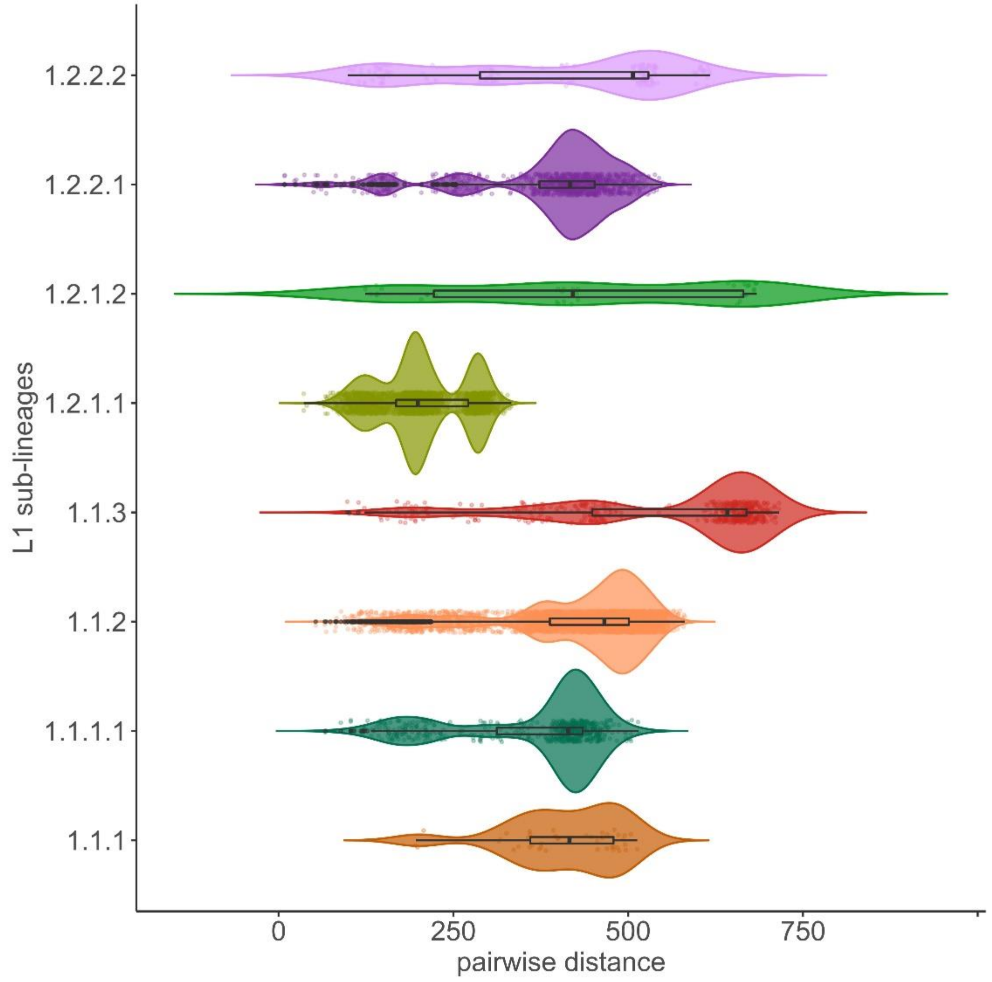

Supplementary Figure 3. Genetic diversity of 312 L1 MTBC strains within sub-lineages. Violin plots show the pairwise SNP distance of strains for each sub-lineage of MTBC Lineage 1. Boxplots within the violins represent the 25th and 75th percentile, black horizontal line the median, black dots are outliers and colored dots the individual pairwise SNP distance values (jitter=0.1).

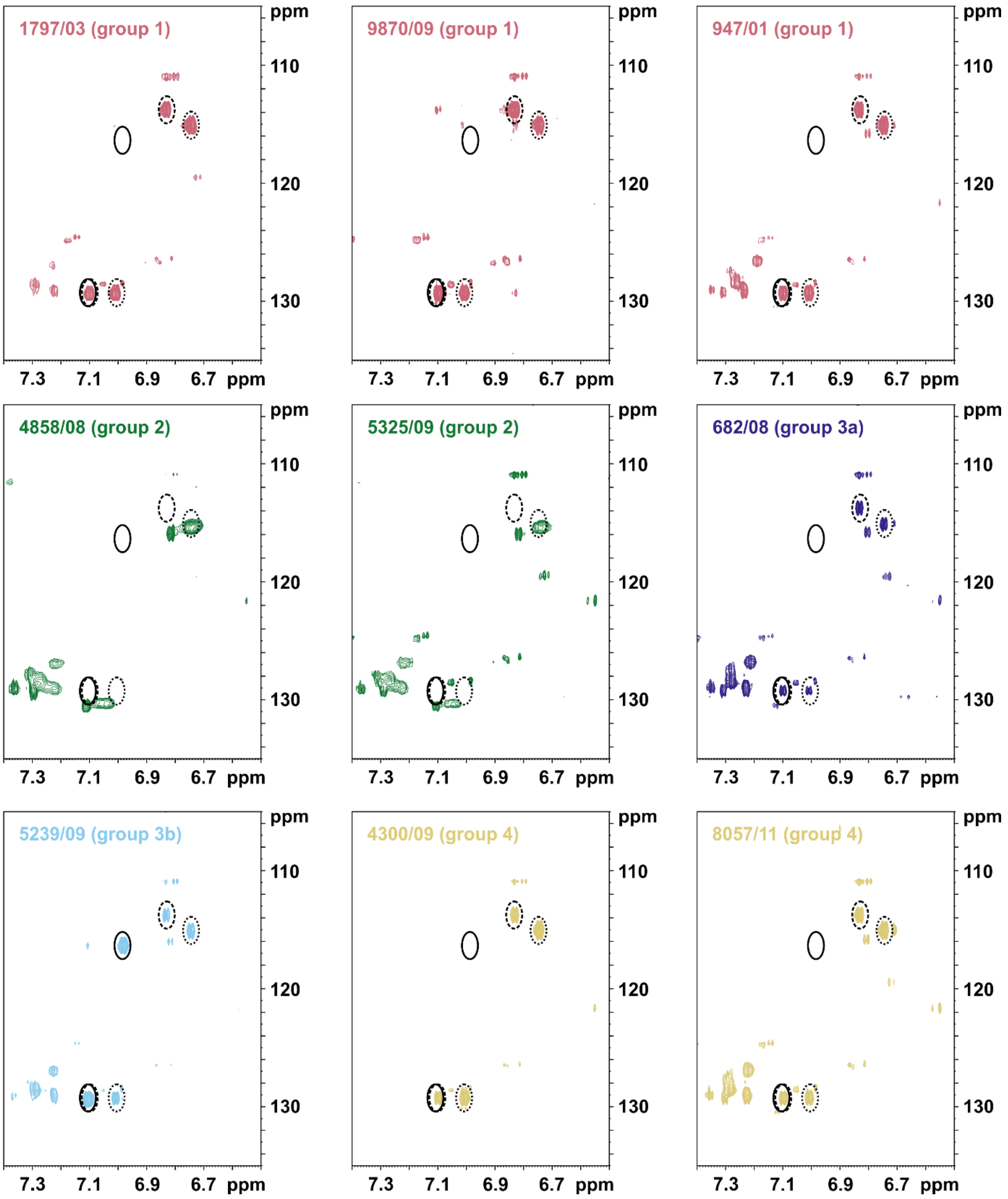

Supplementary Figure 4. Specific signal pattern in $^{1}H,^{13}C$-HSQC NMR maps indicate the presence or absence of phenolphthiocerol dimycoserosates and/or phenolic glycolipids (PGLs; mycoside B/PGL-tb) in total lipid extracts of MTBC L1 clinical isolates (additional strains to Figure 6). The specific chemical shift region (δ_H 7.40-6.50 ppm; δ_C 135-105 ppm) of $^{1}H,^{13}C$-HSQC NMR maps recorded from the total lipid extracts of MTBC L1 strains from different sub-lineages are depicted. The observed C,H-correlations enable a specific determination of the present PGL types[3] in all studied clinical isolates (**Table 2**), for respective chemical structures and further information see **Figure 6**.

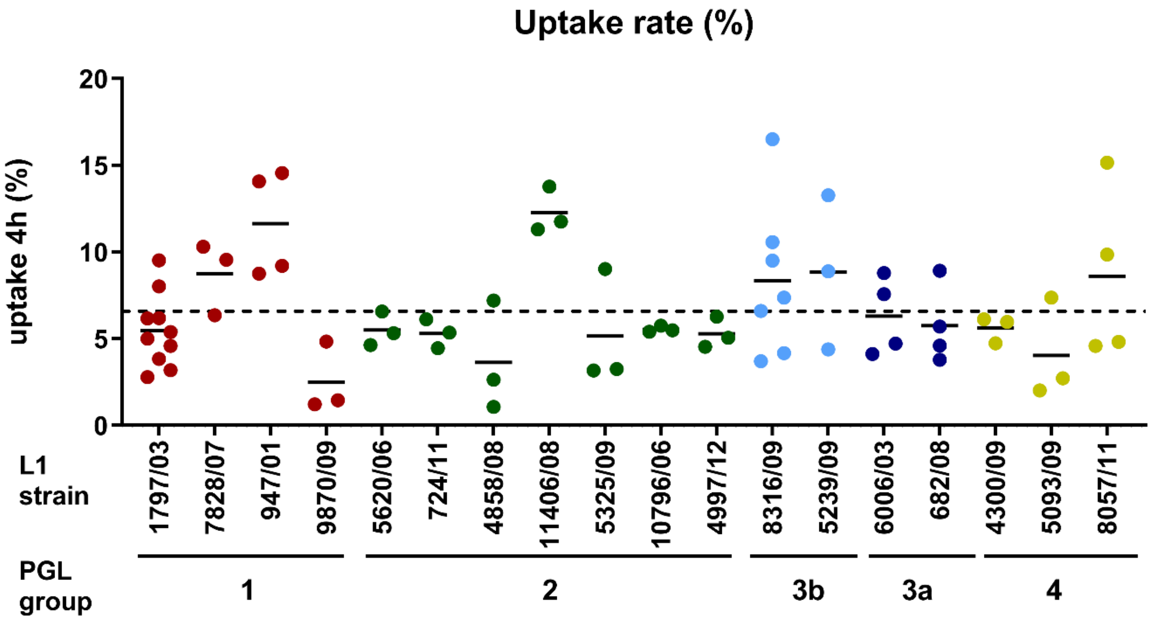

Supplementary Figure 5. Uptake of L1 MTBC strains by human macrophages. Human monocyte-derived macrophages (hMDMs) were infected with strains of different L1 sub-lineages with an MOI of 1:1 for 4 h. Quantification of viable CFU of the inoculum and 4 h post infection was conducted by lysis of monolayers, serial dilution, and plating on 7H10 medium. Shown is the uptake (%) of bacteria related to the inoculum of each individual strain (mean) for at least three independent experiments performed.

Supplementary Tables 1 – 3 are separate data files

Supplementary Table 1: Strain information summary.

Supplementary Table 2: MTBC L1 sub-lineage specific signature SNPs for classification.

Supplementary Table 3: Detected mutations in PGL synthesis genes.

References

1 Coll, F. *et al.* A robust SNP barcode for typing *Mycobacterium tuberculosis* complex strains. *Nature communications* **5**, 4812, doi:10.1038/ncomms5812 (2014).

2 Napier, G. *et al.* Robust barcoding and identification of *Mycobacterium tuberculosis* lineages for epidemiological and clinical studies. *Genome Med* **12**, 114, doi:10.1186/s13073-020-00817-3 (2020).

3 Mahrous, E. A., Lee, R. B. & Lee, R. E. A rapid approach to lipid profiling of mycobacteria using 2D HSQC NMR maps. *Journal of lipid research* **49**, 455-463, doi:10.1194/jlr.M700440-JLR200 (2008).
